# Anticancer Peptides: Design Principles, Translational Bottlenecks, and Emerging Opportunities

**DOI:** 10.3390/molecules31183319

**Published:** 2026-09-19

**Authors:** Marissa E. Di, Yuanpu Peter Di

**Affiliations:** 1 Department of Computational and Systems Biology, University of Pittsburgh School of Medicine, Pittsburgh, PA 15213, USA; dim@pitt.edu; 2 Department of Cellular and Molecular Medicine, Center for Translational Science, Herbert Wertheim College of Medicine, Florida International University, Port St. Lucie, FL 34987, USA

**Keywords:** anticancer peptides, host-defense peptides, peptide therapeutics, peptide engineering, tumor targeting, peptide–drug conjugates, translational development, oncology

## Abstract

Anticancer peptides (ACPs) are frequently discussed as a single therapeutic class, yet the term encompasses molecules that perform markedly different jobs: direct tumor-cell killing, intracellular target modulation, tumor homing and penetration, or selective delivery of a separate payload. This functional diversity creates opportunity, but it also obscures why many potent peptides fail during translation. In this review, we organize ACPs according to their intended pharmacologic role and examine the molecular and product-development principles that determine whether an active sequence can become a useful medicine. Charge, amphipathicity, conformation, target affinity, cellular entry, protease resistance, tissue exposure, and manufacturability must be optimized as an integrated profile rather than as independent attributes. Representative clinical programs—including the oncolytic peptide LTX-315, the cell-penetrating peptide p28, the stapled peptide ALRN-6924, the tumor-penetrating peptide CEND-1, and the cyclic integrin inhibitor cilengitide—illustrate both the reach of peptide pharmacology and recurrent causes of attrition. We propose a developability-centered workflow that links mechanism, route of administration, pharmacokinetics, pharmacodynamics, biomarker strategy, formulation and chemistry, manufacturing, and controls from the beginning of discovery. For the next generation of ACP development, the most credible opportunities lie in route-matched local or regional therapy, mechanism-based combinations, experimentally constrained artificial intelligence, and peptide-enabled delivery systems supported by fit-for-purpose translational models.

## 1. Introduction

Modern oncology increasingly matches treatment to molecular and cellular features, yet therapeutic failure remains common because malignant populations evolve, tumors contain spatially distinct cell states, and effective exposures are often limited by toxicity [1]. Peptides occupy a useful middle ground between small molecules and larger biologics. Their surfaces can engage protein–protein interfaces, their sequences can be modified residue by residue, and their size can permit tissue access that is difficult for antibodies. At the same time, peptides can be rapidly degraded, cleared by the kidney, trapped in endosomes, or neutralized by plasma and extracellular components. The same structural flexibility that makes peptides attractive therefore creates a demanding optimization problem [2,3].

The literature often defines an anticancer peptide by length, source, or in vitro cytotoxicity. These definitions are convenient but insufficient. A nine-residue membranolytic peptide injected into a tumor, a stabilized alpha helix that blocks an intracellular protein–protein interaction, and a cyclic peptide that carries a cytotoxin to a receptor-positive cancer are not interchangeable products. They require different assays, exposure profiles, formulations, safety margins, and clinical endpoints. Treating them as one homogeneous class can lead to inappropriate comparisons and to development programs in which potency is optimized before the intended therapeutic use is defined [4,5].

Prior reviews have classified anticancer peptides by sequence origin, physicochemical properties, mechanism of cell death, cellular target or localization, and therapeutic application. These approaches remain valuable for describing what a peptide is or how it may act. The present framework addresses a different question: what pharmacological job must the peptide product perform, and what route, exposure profile, evidence package, and clinical setting are required for that job? The resulting categories are therefore product-development archetypes rather than a new taxonomy of peptide sequences.

These archetypes are neither mutually exclusive nor defined at a single structural level. A candidate may combine homing, penetration, intracellular modulation, direct oncolysis, or payload delivery. For development purposes, its principal intended pharmacological job should determine the dominant exposure requirement, decisive evidence, and clinical endpoint, while secondary functions should be evaluated as product-specific attributes.

This review adopts a developability-centered perspective. We first distinguish the major pharmacologic roles that peptides can play in oncology. We then examine the physicochemical principles underlying activity and selectivity, followed by the decisions that convert a bioactive sequence into a drug product. Representative clinical experiences are used not as a catalogue of candidates, but as case studies in route selection, patient selection, target validation, and product design. Personalized peptide vaccines are outside the present scope because they function primarily as antigens processed and presented by major histocompatibility complex molecules to prime adaptive T-cell immunity, rather than as direct peptide therapeutic products. Likewise, peptide hormones and peptide receptor radionuclide therapies are treated as adjacent modalities and discussed only when they provide transferable lessons for ACP development.

This article is a focused narrative and critical review. Relevant literature was identified through searches of PubMed, Web of Science, ClinicalTrials.gov, and the reference lists of representative primary studies and reviews through August 2026. Search concepts included anticancer peptides, oncolytic peptides, cell-penetrating peptides, tumor-homing and tumor-penetrating peptides, peptide–drug conjugates, peptide pharmacokinetics and delivery, clinical development, and artificial intelligence-assisted peptide design. Clinical programs were selected to represent distinct pharmacological jobs and informative development outcomes rather than to provide an exhaustive catalogue. Clinical studies were therefore evaluated illustratively and were not screened using a formal systematic review protocol.

## 2. Defining ACPs by Their Pharmacologic Job

**Figure 1:**
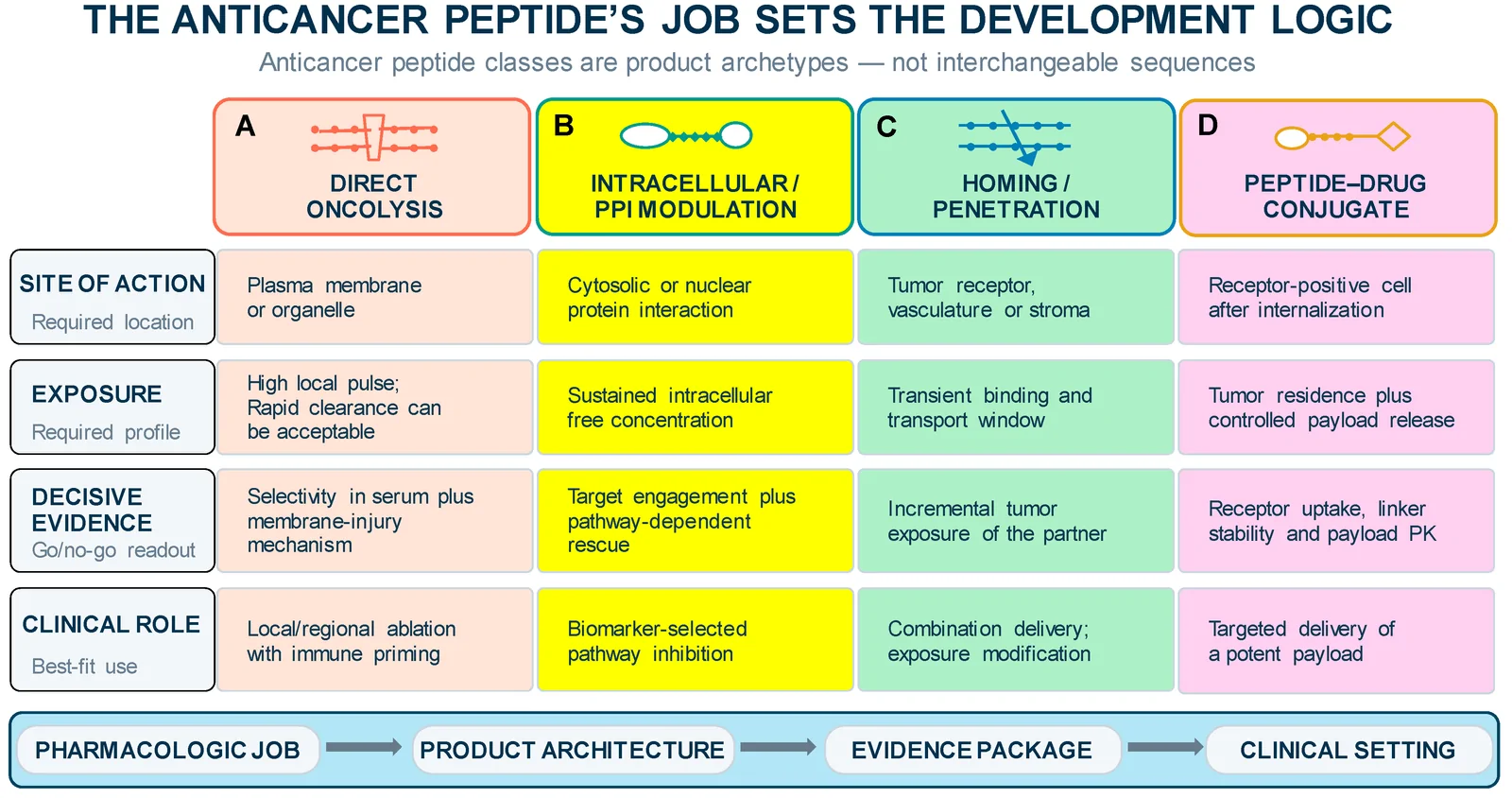
Function-first map of anticancer peptide product archetypes. Anticancer peptides are organized according to their intended pharmacological function: (**A**) direct oncolysis through disruption of cancer-cell or organelle membranes; (**B**) modulation of intracellular targets or protein–protein interactions (PPIs); (**C**) tumor homing or tissue penetration to enhance delivery and exposure; and (**D**) peptide–drug conjugation for receptor-mediated delivery of a therapeutic payload. For each archetype, the map links the required site of action and exposure profile to the decisive experimental evidence and most appropriate clinical role. These categories are product-development archetypes rather than interchangeable sequence classes; therefore, each requires a distinct molecular architecture, pharmacokinetic profile, validation strategy, and clinical setting. The bottom workflow emphasizes a function-first development principle in which the pharmacological job defines the product architecture, evidence package, and intended clinical application. PK, pharmacokinetics; PPI, protein–protein interaction. This figure presents overlapping product-development archetypes rather than a rigid classification system. The high-resolution vector graphic uses redundant labels and a color-blind-aware palette so that interpretation does not depend on color alone.

The four product-development archetypes and their principal evidence requirements are summarized in Figure 1 and Table 1.

### 2.1. Direct Tumoricidal and Oncolytic Peptides

Many ACPs originated from antimicrobial or host-defense peptides and retain cationic, amphipathic architectures [6,7]. They can bind cancer-cell membranes, insert into lipid bilayers, destabilize organelles, or initiate regulated and non-regulated cell death. Their principal advantage is kinetic: membrane injury can occur within minutes and does not require a proliferating cell or an intact apoptotic pathway. This offers a plausible route around some mechanisms of multidrug resistance. Their principal liability is also mechanistic. A peptide that kills by interacting with lipid bilayers must discriminate among tumor cells, erythrocytes, activated leukocytes, vascular endothelium, and other exposed host membranes under realistic concentrations and exposure times.

Altered anionic phospholipid exposure is one contributor to preferential membrane interaction, but cancer selectivity may also reflect lipid composition and organization, surface-charge distribution, membrane potential, glycocalyx architecture, serum and matrix binding, vascular exposure, and the local tissue context. These features are heterogeneous and must be tested rather than assumed.

Directly oncolytic peptides may be most tractable when local exposure is intended. LTX-315, a nine-residue peptide developed from lactoferricin-related chemistry, illustrates this product concept. Intratumoral administration produces local membrane disruption and can release tumor antigens and danger signals. In a phase I study, LTX-315 increased intratumoral CD8-positive T-cell infiltration and produced substantial shrinkage in a subset of injected lesions, while systemic and hypersensitivity reactions emphasized that local administration does not eliminate safety considerations [8,9]. The program shows how route, mechanism, and immune pharmacodynamics can be integrated into a single therapeutic strategy.

In this framework, immune modulation is treated as a mechanism-dependent pharmacodynamic consequence or combination opportunity—for example, following local oncolysis and antigen release—rather than as a fifth mutually exclusive product archetype.

### 2.2. Intracellular and Protein-Interaction Modulators

Peptides can reproduce short recognition motifs that mediate protein–protein interactions, including interfaces that are comparatively flat and difficult for conventional small molecules. However, high affinity in a biochemical assay is only the first requirement. Intracellular peptides must cross the plasma membrane, escape endosomes when uptake is vesicular, reach the correct subcellular compartment, resist proteolysis, and remain sufficiently soluble to avoid non-specific binding. Total cellular uptake should not be equated with productive intracellular delivery: cytosolic or organellar availability, endosomal escape, and target engagement require separate quantitative evidence.

Structural constraint is especially valuable for this class. Hydrocarbon stapling can stabilize an alpha helix, enhance protease resistance, and sometimes improve cellular uptake. The seminal demonstration that a stapled BH3 helix could reactivate apoptosis in vivo established the approach, while subsequent work showed that constraint geometry and staple placement can either preserve or impair function [10,11].

The azurin-derived peptide p28 provides one clinical model. P28 is a cell-penetrating peptide that interacts with the p53 regulatory network through a mechanism distinct from direct membrane lysis. An initial phase I trial in patients with advanced solid tumors reported acceptable tolerability and evidence of biologic activity [12]. Such findings are encouraging, but they also underscore a general problem in early peptide trials: broad eligibility can establish safety while providing limited information about which tumors are most dependent on the targeted pathway.

ALRN-6924 (sulanemadlin), a stabilized alpha-helical peptide that inhibits MDM2 and MDMX, provides another model and advanced into phase I testing in TP53-wild-type tumors and lymphomas. Its clinical development demonstrates that a peptide can achieve systemic intracellular pharmacology, but also that genotype, exposure, target engagement, and schedule must remain tightly aligned [13]. Subsequent development of the original systemic strategy was curtailed, although the molecule has since been considered for local intraocular evaluation in retinoblastoma [14].

### 2.3. Tumor-Homing and Tissue-Penetrating Peptides

Some peptides are not intended to kill cancer cells. Their pharmacologic job is navigation. Tumor-homing peptides bind receptors on malignant cells, stroma, or tumor vasculature; tissue-penetrating peptides can activate transport pathways that increase access beyond the vascular compartment. The C-end rule (CendR) pathway, in which peptides bearing an exposed C-terminal (R/K)XX(R/K) motif engage neuropilin-1, is a well-characterized example [15]. iRGD combines integrin binding, proteolytic activation, and neuropilin-1-dependent penetration, and preclinical work showed that it can enhance intratumoral delivery of co-administered agents [16].

This enabling role changes the development question. The relevant endpoint is not necessarily the peptide’s plasma half-life or cytotoxicity, but whether it reproducibly improves tumor exposure to the partner therapy without increasing normal-tissue exposure. CEND-1 (certepetide), a cyclic tumor-penetrating peptide, was evaluated with gemcitabine and nab-paclitaxel in metastatic pancreatic ductal adenocarcinoma. The phase I study reported no dose-limiting toxicities attributed to the combination and encouraging activity, supporting further randomized evaluation [17]. Because the peptide is an exposure modifier, its clinical value must ultimately be measured against the same backbone regimen without the peptide. Subsequent randomized phase II ASCEND meeting reports showed cohort-dependent signals of incremental benefit, but full peer-reviewed confirmation and definitive phase III validation remain necessary [18].

### 2.4. Cargo-Bearing Peptides and Peptide–Drug Conjugates

Peptide–drug conjugates (PDCs) combine a targeting or uptake sequence, a linker, and a cytotoxic or imaging payload. Their performance depends on the complete molecular assembly. Receptor affinity that is too high may restrict tissue distribution; a linker that is too stable can prevent intracellular release, whereas premature cleavage can recreate the systemic toxicity of the free payload. Payload hydrophobicity may alter peptide solubility, aggregation, plasma protein binding, and clearance. For PDCs, the conjugate—not the unconjugated targeting peptide—is the drug [19]. Conjugation can also change receptor affinity and internalization, hydrodynamic behavior, tissue penetration, bystander exposure, and the therapeutic index; these properties must therefore be re-established for each peptide-linker-payload assembly.

Adjacent peptide-targeting modalities provide important proof of principle. In somatostatin receptor (SSTR)-positive neuroendocrine tumors, lutetium-177-DOTATATE couples the somatostatin analogue octreotate—which targets SSTRs, particularly SSTR2—to a beta-emitting radionuclide and improved progression-free survival in the NETTER-1 trial [20]. This therapy is not a direct ACP, but it demonstrates the clinical power of pairing receptor-defined patient selection, companion imaging, dosimetry, and a peptide-guided payload. ACP developers should treat this integrated diagnostic–therapeutic architecture as a model rather than assuming that peptide targeting alone guarantees selectivity. Peptide receptor radionuclide therapy is included here as an adjacent proof-of-principle modality, not as a direct ACP archetype. Its transferable lesson is that peptide targeting becomes clinically actionable when receptor expression, companion imaging, whole-body distribution, tumor heterogeneity, and payload-specific dosimetry are integrated into patient selection and treatment planning.

## 3. Molecular Principles Governing Activity and Selectivity

ACP activity and developability emerge from a coupled physicochemical profile that includes length, net charge and charge distribution, hydrophobicity and hydrophobic moment, amphipathicity, conformational propensity, solubility, aggregation, chemical and proteolytic stability, and permeability. These variables should not be optimized toward a universal maximum. Their preferred ranges depend on the peptide’s intended pharmacological job, route, site of action, and required duration of free exposure; improving one property may worsen another, such as increasing membrane activity while also increasing hemolysis, aggregation, or nonspecific tissue binding.

### 3.1. Charge and Amphipathicity Are Context Dependent

Cancer cells can display increased anionic character through phosphatidylserine exposure, altered glycosylation and sialylation, and changes in membrane composition [6]. Anionic phospholipids can also be exposed on tumor endothelium [21,22]. These features provide a rationale for cationic ACPs, but they are neither uniform across tumors nor absent from normal physiology. Activated and apoptotic cells, extracellular vesicles, and injured tissues may present similar surfaces. Moreover, albumin, lipoproteins, glycosaminoglycans, salts, and cell density can substantially change peptide activity between a simple culture assay and a tumor.

Net charge, hydrophobicity, hydrophobic moment, and secondary-structure propensity jointly influence binding and insertion [6,23]. Increasing cationicity can improve initial attraction but can also increase non-specific uptake and tissue binding. Increasing hydrophobicity can strengthen membrane disruption while worsening hemolysis, aggregation, and formulation. Consequently, the appropriate objective is not maximal cytotoxicity. It is a reproducible therapeutic window under conditions that approximate the intended route and exposure.

### 3.2. Cell Death Mechanism Shapes Combination and Biomarker Strategy

Membrane lysis, mitochondrial permeabilization, apoptosis, necrosis, autophagy-related death, and other stress responses are often reported as interchangeable evidence of ACP activity. They are not equivalent clinically. Rapid lysis may be desirable for intratumoral immune priming but unacceptable after systemic administration. A mitochondrial peptide may synergize with a BCL-2-family inhibitor, whereas a peptide that disrupts DNA-repair complexes may be better paired with radiation or a DNA-damaging agent. The amount and location of cell death also influence antigen release, dendritic-cell activation, vascular injury, and local inflammation [9,24].

Mechanism should therefore be demonstrated with orthogonal assays and linked to a falsifiable combination hypothesis. Claims of “immunogenic cell death” should include evidence for relevant danger signals, antigen-presenting-cell activation, and adaptive immune contribution, rather than relying on one biomarker. Likewise, resistance claims should be tested in models with defined resistance mechanisms and compared at clinically plausible exposures.

Mechanistic assignment should be matched to pharmacodynamic evidence: membrane disruption to time-resolved permeability and structural injury; mitochondrial action to organelle-localized exposure, depolarization, or cytochrome-c release; apoptosis to complementary caspase, substrate-cleavage, and rescue evidence; and immunogenic cell death to a pattern of danger-signal release, antigen-presenting-cell activation, and immune-dependent antitumor activity. No single marker is sufficient to establish a complex cell-death program.

Systemic activity of a host-defense-like lytic peptide in prostate and breast xenografts provided an important proof of concept, but such models also emphasize the need to distinguish antitumor exposure from tolerability margins that may differ in humans [25].

### 3.3. Conformation Controls Both Pharmacology and Developability

Linear peptides are conformationally flexible and may pay a large entropic penalty on binding. Cyclization, stapling, N-methylation, disulfide engineering, incorporation of non-canonical or D-amino acids, and half-life-extension chemistries can preorganize a binding surface or resist degradation [26,27,28,29]. Yet each modification can change more than one property. Cyclization may reduce degradation but also reduce solubility; D-amino-acid substitution may preserve membrane activity but disrupt stereospecific receptor binding; and lipidation may extend exposure while increasing plasma-protein binding.

This multidimensional behavior argues for matched analog panels rather than serial one-property optimization. Potency, selectivity, solubility, aggregation, protease stability, permeability, and exposure should be measured in parallel as early as material permits. A modification should advance only when the resulting profile supports the intended product—not simply because one assay improves. Because those conformational choices determine not only pharmacology but also route, exposure, formulation, and safety, they must now be translated into an explicit target product profile.

## 4. Developability by Design: Converting a Sequence into a Product

### 4.1. Start with a Target Product Profile

Peptide discovery often begins with an active sequence and postpones the clinical-use question. Reversing that order improves decision quality. A minimal target product profile should specify the cancer setting, intended route, dosing frequency, monotherapy or combination role, required duration of exposure, acceptable local and systemic toxicity, patient-selection strategy, and a plausible comparator. These choices determine whether rapid clearance is a defect or an advantage. For an intratumoral oncolytic peptide, brief local exposure may be sufficient; for a systemic PPI inhibitor, sustained free plasma and intracellular concentrations may be essential.

The target product profile also prevents delivery technology from becoming an automatic response to weak pharmacology. Nanoparticles, polymers, exosomes, and depot formulations can protect peptides, but they create new distribution, clearance, manufacturing, and regulatory questions. A carrier should solve a demonstrated exposure problem and be compared with simpler alternatives, including sequence stabilization, albumin binding, conjugation, infusion, or local delivery.

### 4.2. Build a Stage-Gated Assay Cascade

Discovery assays should distinguish true activity from artifacts caused by aggregation, detergent-like behavior, plate binding, or unusually permissive media. Direct cytotoxic ACPs should be evaluated across tumor cells, matched non-malignant cells, primary human cells, erythrocytes, and immune populations. Time-kill kinetics, washout experiments, serum shifts, and cell-density effects are often more informative than a single 24- or 72-h half-maximal inhibitory concentration. Intracellular inhibitors require quantitative uptake, endosomal escape, target engagement, and rescue or resistance experiments that connect phenotype to the intended target.

Library technologies should be chosen to match the intended chemical space. Phage-encoded bicyclic libraries, for example, can combine the throughput of biological display with chemical constraint and have expanded access to high-affinity cyclic binders [30]. Whatever the discovery platform, activity measurements should be accompanied by explicit counter-screens and sequence-diversity controls.

Early developability measurements should include aqueous solubility across relevant pH, aggregation, chemical stability, plasma and tissue protease stability, microsomal or lysosomal liability where relevant, plasma-protein binding, and whole-blood compatibility. The sequence should then face an explicit advancement rule. For example, a systemic membranolytic ACP should not advance on potency alone if its hemolytic or primary-cell selectivity collapses in the concentration range needed for in vivo exposure.

Aggregation should be assessed across the concentration range, formulation, temperature, time, and biological matrices relevant to dosing. For cationic or amphipathic peptides, self-association can either reduce the freely available concentration or create particles with exaggerated membrane disruption, nonspecific uptake, protein binding, altered clearance, and tissue sequestration. Apparent activity may therefore change with plate material, cell density, serum, mixing, or dosing concentration. No single assay is sufficient; complementary approaches may include turbidity or light scattering, dynamic light scattering, size-exclusion chromatography, analytical ultracentrifugation, microscopy, and recovery of soluble peptide, selected according to peptide size and formulation.

**Figure 2:**
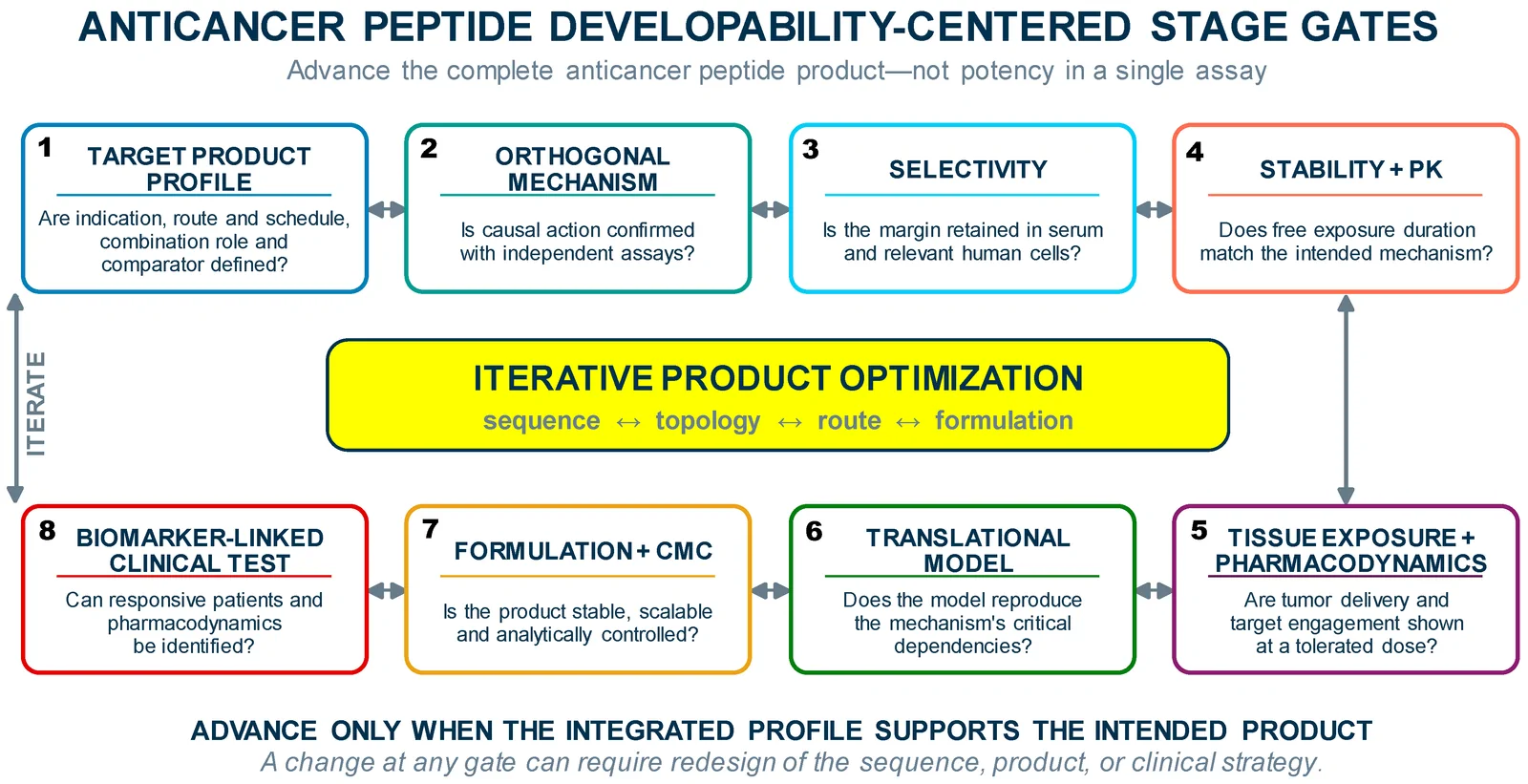
Developability-centered stage-gate framework for anticancer peptide advancement. Candidate products progress through eight interconnected decision gates: (1) definition of the target product profile; (2) confirmation of the proposed mechanism using orthogonal assays; (3) demonstration of selectivity in serum and relevant human cells; (4) alignment of stability and pharmacokinetics with the intended mechanism; (5) confirmation of tissue exposure, target engagement, and pharmacodynamic activity at a tolerated dose; (6) validation in translational models that reproduce critical mechanistic dependencies; (7) establishment of a stable, scalable, and analytically controlled formulation and manufacturing process; and (8) development of a biomarker-linked clinical testing strategy. The bidirectional arrows indicate that development is iterative rather than strictly linear. Findings at any gate may require redesign of the peptide sequence, molecular topology, administration route, formulation, or clinical strategy. Advancement should therefore be based on the integrated product profile rather than potency in a single assay. CMC, chemistry, manufacturing, and controls; PK, pharmacokinetics. The high-resolution vector graphic uses numbered gates, text labels, and a color-blind-aware palette so that the workflow remains interpretable without color discrimination.

**Scheme 1:**
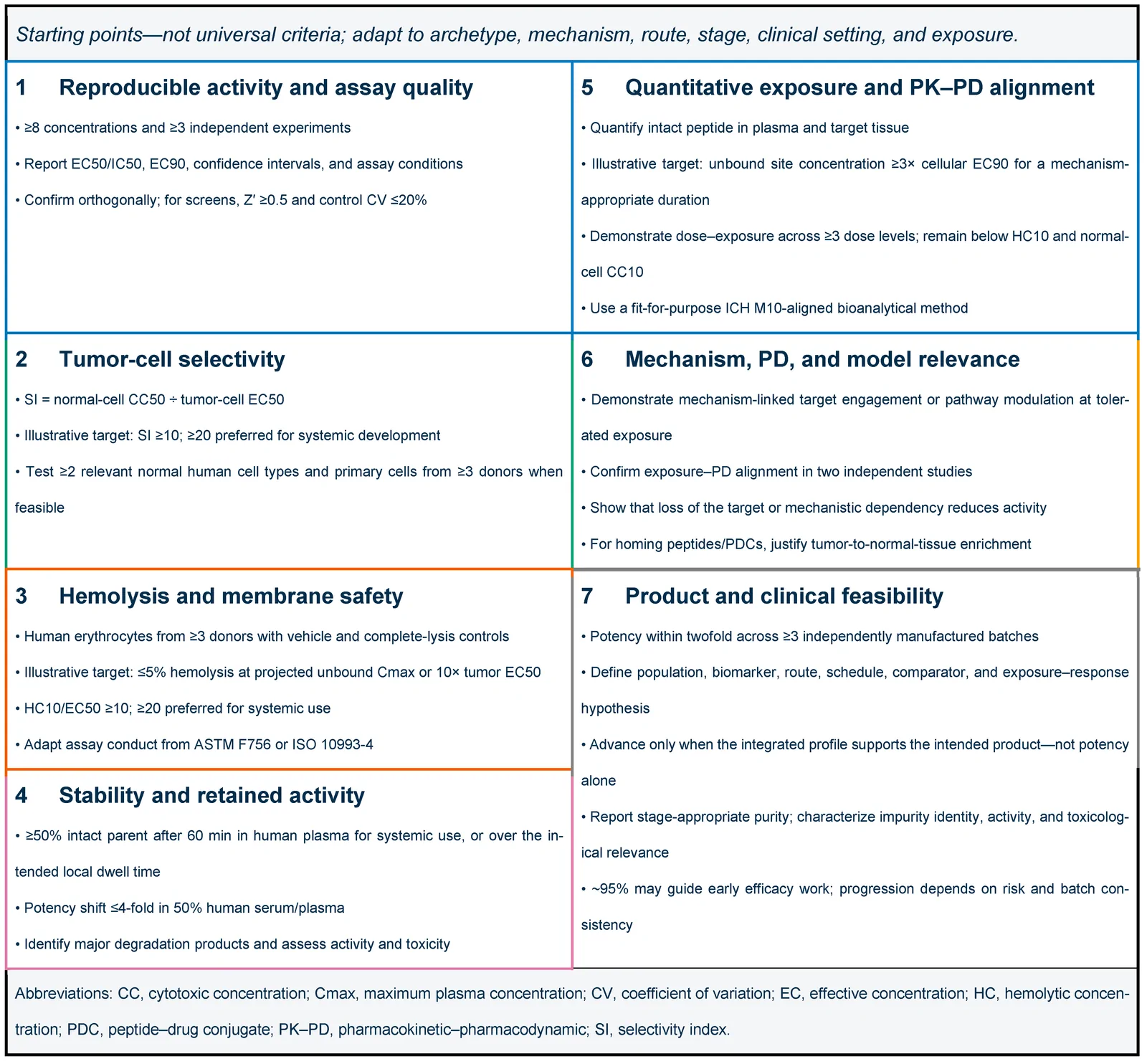
Illustrative early translational benchmarks for anticancer peptide development before efficacy expansion. Standards cited for the assays named in the box are ASTM F756 and ISO 10993-4 [31,32], and ICH M10 [33].

The resulting iterative development logic is summarized in the stage-gate framework shown in Figure 2 and operationalized through the illustrative benchmarks in Scheme 1.

### 4.3. Match Stability Engineering to the Required Exposure

Common peptide half-life extension strategies include terminal protection, D-amino-acid substitution, cyclization, N-methylation, stapling, PEGylation, lipidation, fusion to larger carriers, and reversible albumin binding [26,27,28,29]. The optimal strategy depends on where the pharmacologic target resides. Protection from exopeptidases may be sufficient for a locally administered peptide, whereas a systemically delivered intracellular inhibitor may require resistance to endopeptidases, prolonged circulation, and efficient tissue and cellular entry.

Albumin-binding approaches can extend exposure without permanently enlarging the peptide; one biomimetic strategy used a transthyretin-binding moiety to recruit serum protein and prolong peptide half-life [28]. Such approaches can be valuable, but total plasma concentration should not be confused with free pharmacologically available concentration. Distribution and target engagement must be measured directly.

### 4.4. Treat Route and Formulation as Part of Mechanism

Systemic intravenous dosing is not the default endpoint for every ACP. Intratumoral, intra-arterial, intraperitoneal, inhaled, topical, and implantable-depot routes can generate high regional concentrations for targeted tumors and reduce systemic exposure. Route selection should reflect disease anatomy and clinical workflow. Accessible lesions may permit repeated injection; lung or pleural malignancies may be candidates for regional delivery; postoperative cavities may support local depots. These strategies can convert a narrow systemic therapeutic window into a useful local product, provided local tissue injury and procedural feasibility are rigorously assessed.

For systemic delivery, carriers should be selected by function: protection from proteases, control of release, receptor-mediated targeting, or tissue penetration. Reliance on the enhanced permeability and retention effect alone is risky because nanoparticle entry and distribution in human tumors are heterogeneous, and non-vascular transport mechanisms can dominate [34]. The relevant comparison is not carrier versus free peptide at an arbitrary dose; it is whether the carrier improves exposure at the site of action and therapeutic index at matched systemic toxicity.

### 4.5. Design for Manufacturing and Control

Chemical complexity accumulates quickly. A peptide containing multiple non-canonical residues, two staples, a cleavable linker, a targeting ligand, and a nanoparticle formulation may be scientifically elegant but difficult to characterize and reproduce. Sequence-related impurities, epimers, deletion products, oxidation, deamidation, aggregation, and linker or payload variants require analytical methods and justified specifications. Longer sequences and extensive modification can reduce synthesis yield and increase solvent use, purification burden, and cost [35,36].

Manufacturability should therefore be assessed during lead optimization. The minimum effective length, number of stereocenters and modifications, feasible solid-phase or recombinant route, purification recovery, formulation stability, container compatibility, and scale-dependent impurity profile should be known before late preclinical development. Quality by design is especially important for PDCs, for which heterogeneity can arise from the peptide, linker, payload, and conjugation process [37].

Molecular quality cannot be inferred from a single chromatographic purity value. Depending on the sequence and architecture, development methods should distinguish deletion products, epimers, oxidation and deamidation products, disulfide-scrambled or regioisomeric species, aggregates, counterion forms, residual coupling or cleavage reagents, and linker- or payload-related variants. Orthogonal mass, chromatographic, spectroscopic, and functional methods are therefore needed to connect impurity identity with biological activity and toxicological risk.

The numerical examples are decision-support starting points rather than regulatory acceptance thresholds. Hemolysis and blood-compatibility procedures may be adapted from ASTM F756 and ISO 10993-4, and exposure measurements should use fit-for-purpose bioanalytical methods consistent with ICH M10; these standards guide assay conduct but do not define universal ACP-specific progression criteria.

## 5. Translational Lessons from Representative Programs

Clinical history supports neither blanket optimism nor pessimism. Peptides have reached tumors, engaged intracellular targets, altered immune infiltrates, and delivered potent payloads. They have also failed in randomized trials despite strong biologic rationale. The most useful question is what each program teaches about the fit among mechanism, product design, and clinical setting.

Representative programs were selected not merely as examples of peptide diversity but as case studies in the alignment—or misalignment—of therapeutic hypothesis, route, exposure, biomarker strategy, pharmacodynamic evidence, and development outcome.

LTX-315 demonstrates the value of a route-matched product. Intratumoral delivery concentrates a membranolytic peptide where acute lysis and inflammation are desired. Its phase I data support biological activity but also show that lesion-level and immune effects do not automatically translate into conventional objective responses [9]. Future trials of local ACPs should prospectively define injected-lesion response, non-injected-lesion response, immune pharmacodynamics, and the contribution of combination immunotherapy.

p28 and ALRN-6924 demonstrate that cell-penetrating and structurally stabilized peptides can be administered systemically and interrogate intracellular cancer biology [12,13]. Their development also highlights the importance of molecular selection. A p53-pathway therapy needs a biomarker strategy that goes beyond a nominal TP53 label and addresses pathway competence, negative regulators, target dependence, and intratumoral exposure.

CEND-1 shows that a peptide can be developed as an enhancer of a standard regimen rather than as an independent cytotoxin [17]. The decisive test is randomized evidence that the peptide adds benefit to the backbone therapy. Cilengitide, a cyclic RGD peptide targeting alpha-v beta-3 and alpha-v beta-5 integrins, provides complementary caution. Despite encouraging early studies, adding cilengitide to standard chemoradiotherapy did not improve outcomes in the phase III CENTRIC trial [38]. The result does not invalidate cyclic peptides; it shows that target expression, schedule, tumor biology, and disease selection must support the proposed mechanism.

For enabling peptides such as CEND-1, encouraging single-arm activity is hypothesis-generating; the decisive evidence is randomized incremental benefit over the same backbone regimen. For intracellular programs such as ALRN-6924, demonstration of systemic exposure and pathway pharmacodynamics is an important achievement, but it does not by itself ensure successful indication selection, schedule, therapeutic index, or continued development.

Representative clinical programs, current outcomes, and transferable development lessons are compared in Table 2, while their publicly disclosed sequences or structural formats and key structure–property implications are summarized in Table 3.

## 6. Challenges That Should Reset Field Standards

### 6.1. Selectivity Must Be Demonstrated at Achievable Exposure

Cancer-selective activity is often reported as a ratio of half-maximal effects in one cancer line and one immortalized normal line. This is inadequate for membrane-active peptides and misleading for targeted peptides if the comparator lacks the target. Selectivity should be established across a panel representing the tissues most exposed by the intended route and at concentrations supported by pharmacokinetics. Whole-blood compatibility, complement activation, cytokine release, vascular effects, and repeat-dose immune responses should be incorporated when the peptide’s charge, aggregation, or origin makes those risks plausible.

Selectivity has several non-interchangeable dimensions. Molecular selectivity describes preferential binding to the intended target; cellular selectivity compares effects in target-positive malignant cells with relevant normal cells under matched conditions; tissue selectivity reflects spatial exposure and retention in tumor versus vulnerable normal organs; pharmacokinetic selectivity reflects differences in free, active concentration over time; and therapeutic selectivity is the integrated separation between antitumor benefit and clinically relevant toxicity. A favorable result at one level does not establish selectivity at the others.

### 6.2. Tumor Heterogeneity Is a Distribution Problem as Well as a Target Problem

Receptor abundance varies among patients, lesions, and regions within a lesion. Dense matrix, high interstitial pressure, necrosis, abnormal vasculature, and binding-site barriers can create steep concentration gradients. Bulk tumor concentration may therefore conceal inadequate exposure of viable target cells. Spatial pharmacology—using imaging, microdissection, autoradiography, or spatial omics—should become routine for targeted and conjugated peptides. A successful product needs sufficient exposure in the cells that drive progression, not merely detectable accumulation in the tumor mass.

Tumor accumulation is only the first distribution measure and should not be equated with productive delivery. A peptide detected in a bulk tumor sample may remain intravascular, bind extracellular matrix, accumulate in necrotic regions, enter non-target stromal cells, remain trapped in endosomes, or persist as inactive degradation products. Productive delivery requires mechanism-appropriate penetration into viable tumor regions, access to the relevant cell population and subcellular compartment, release of an active payload where applicable, target engagement, and a downstream pharmacodynamic response. These steps can be evaluated by spatial imaging or autoradiography, microdissection and quantitative mass spectrometry, cellular or subcellular fractionation, release assays, direct target-engagement methods, and mechanism-linked pharmacodynamic markers.

Potency, exposure, target engagement, pharmacodynamic activity, and efficacy are sequential but non-equivalent concepts. Potency is an assay-defined concentration-effect relationship; exposure is the concentration-time profile of intact, biologically available product at the relevant site; target engagement demonstrates physical interaction with or occupancy of the intended target; pharmacodynamic activity demonstrates the predicted downstream biological response; and efficacy is the resulting therapeutic benefit. Total tumor concentration can include plasma-confined, protein-bound, matrix-bound, degraded, receptor-sequestered, or endosome-trapped material and therefore may not represent free peptide in the pharmacologically relevant compartment.

### 6.3. Preclinical Models Must Reproduce the Mechanism’s Dependencies

Subcutaneous xenografts can overestimate exposure and cannot model an intact immune response. Syngeneic models preserve immunity but may not reproduce the human target or peptide metabolism. Genetically engineered models offer native tissue architecture but are slower and can be less suitable for screening. Patient-derived organoids and ex vivo tumor cultures can preserve clinically relevant genotypes and, in some formats, immune and stromal components [42,43]. No single model is sufficient. Model selection should be based on the mechanism’s dependencies: membrane composition for a lytic peptide, receptor distribution for a homing peptide, immune competence for an immunogenic therapy, and human protease stability for a systemic product.

### 6.4. Dose Finding Should Optimize Benefit-Risk, Not Merely Identify Tolerance

Peptide pharmacology may be transient, saturable, locally concentrated, or schedule dependent. The highest tolerated dose can be biologically unnecessary and may worsen non-specific effects. Early trials should integrate exposure, target engagement, lesion pharmacodynamics, and repeated sampling to identify an optimized dose and schedule. For oncology drugs and biological products within its scope, current FDA guidance emphasizes dose optimization rather than automatic selection of the maximum tolerated dose [44]; analogous mechanism-appropriate principles should be applied cautiously to peptide modalities outside that guidance. For local peptides and PDCs, route-specific or payload-specific dose metrics may be required.

## 7. Future Opportunities

### 7.1. Route-First Development Can Rescue Valuable Mechanisms

The most immediate opportunity is not a new chemistry but better matching of peptide pharmacology to anatomy. Local and regional administration can exploit rapid action while limiting systemic exposure. Intratumoral peptides can combine tumor debulking with immune priming; inhaled or airway-directed formulations could provide high regional exposure for selected pulmonary malignancies; postoperative depots could target residual disease at a defined site. These approaches require dedicated toxicology and formulation, but they may offer a shorter path than forcing every active sequence into chronic systemic dosing.

### 7.2. Combinations Should Be Mechanism-Led

Peptides are well suited to combinations when they alter access or cell state. Tumor-penetrating peptides can increase delivery of a partner drug; membranolytic peptides can release antigens and inflammatory signals; and mitochondrial or signaling peptides can lower the threshold for chemotherapy or radiation. The field should move from descriptive synergy matrices to designs that identify the order, timing, and pharmacodynamic consequence of each agent. The peptide’s contribution must remain measurable, particularly when the partner regimen is already active.

### 7.3. Artificial Intelligence Should Optimize Constrained, Testable Profiles

Machine learning can prioritize sequences, predict activity or toxicity, and generate candidate binders. Recent studies have combined generative models with docking and molecular dynamics to produce experimentally improved peptide inhibitors of beta-catenin and NEMO, while protein-language-model approaches have generated target-conditioned peptide binders [45,46]. Structure-aware methods are also expanding cyclic-peptide design [47]. These are meaningful advances, but they do not remove the need for pharmacology. Models trained on heterogeneous cytotoxicity labels can reproduce assay bias, and random data splitting can exaggerate performance when homologous sequences appear in training and test sets.

Training-set quality is therefore a central constraint. Many ACP datasets combine positives measured in different cell lines, media, serum conditions, exposure times, and endpoint assays, whereas ‘negative’ sequences may simply lack an ACP annotation or may have been drawn from unrelated proteins rather than shown experimentally to be inactive. A model can then learn length, charge, hydrophobicity, sequence source, or laboratory-specific assay signatures instead of transferable anticancer biology. The field needs standardized negative datasets: peptides tested alongside positives under the same protocols and confirmed inactive at prespecified concentrations, plus hard negatives matched for length, net charge, hydrophobicity, and structural class that fail on efficacy or selectivity. Each negative should carry assay metadata, peptide purity, aggregation and solubility information, serum conditions, normal-cell toxicity, and detection limits. Sequence-similarity-aware family splits should keep close analogues out of both training and test sets, and models should undergo blinded external validation on balanced panels. Existing ACP predictors illustrate the dependence on curated positive and non-ACP sets [48]. A concrete example is the widely reused alternate AntiCP 2.0 benchmark, which paired 970 experimentally validated ACPs with 970 peptides randomly sampled from Swiss-Prot [49]. Because these negative sequences were not experimentally confirmed to lack anticancer activity and may differ systematically in source or composition, a model can learn dataset construction rather than ACP biology. Systematic peptide benchmarking further shows that negative-sampling strategy alone can substantially bias reported performance [50].

The most productive near-term role of AI is not isolated prediction of anticancer activity, but transparent multi-objective ranking against prespecified product requirements. Models should prioritize candidates across potency, cellular and blood compatibility, aggregation, solubility, chemical and proteolytic stability, permeability, target engagement, exposure, and manufacturability. For translational use, performance should be reported separately for each endpoint, evaluated against withheld sequence families, and confirmed using standardized positive and negative panels under matched assay conditions. This emphasis on benchmark quality and prospective validation prevents a favorable composite score from obscuring failure in a decisive safety, exposure, or manufacturability criterion.

### 7.4. Peptide-Enabled Delivery May Mature Faster than Standalone Systemic Cytotoxicity

Homing peptides, penetration peptides, PDCs, and radioligands can exploit high-affinity recognition while assigning cell killing to a payload with established potency. This division of labor may be more developable than requiring one short sequence to provide targeting, penetration, stability, and cytotoxicity simultaneously. The opportunity is strongest when the target can be measured clinically and when imaging or a circulating biomarker can confirm engagement. Modular platforms should nevertheless avoid assuming that a targeting sequence is portable across payloads; every new linker–payload combination changes the molecule’s distribution and safety.

### 7.5. Translation-Ready Evidence Packages Can Reduce Avoidable Attrition

The field would benefit from common reporting standards that connect sequence, purity, counterion, formulation, assay conditions, exposure, and outcome. Negative results—loss of activity in serum, hemolysis, poor tumor penetration, or failure of a stabilizing modification—are particularly valuable for computational design and should be captured in interoperable datasets. Academic programs can improve their translational readiness by producing a concise data package aligned with Scheme 1 before expanding efficacy claims. This would make partnership decisions more evidence-based and allow promising ACPs to be compared on developability rather than on in vitro potency alone.

## 8. Conclusions

Anticancer peptides are not one modality. They are a family of molecular formats that can kill, inhibit, navigate, penetrate, or carry cargo. Their future depends less on discovering ever more cytotoxic sequences than on defining the peptide’s therapeutic job and designing the entire product around it. Mechanism, route, exposure, biomarker, formulation, manufacturing, and clinical endpoint must be connected from the start.

Clinical experience already provides a practical roadmap. Local oncolytic peptides show how route can create a therapeutic window; stabilized helices show that intracellular peptide pharmacology is achievable; tumor-penetrating peptides show how a peptide can enable another medicine; and negative randomized trials show the cost of weak biological selection. In the future, the strongest ACP programs will be those that combine chemical ingenuity with disciplined development: fit-for-purpose models, quantitative pharmacology, scalable chemistry, and trials capable of testing the mechanism. With that shift, selected peptides can progress from compelling sequences to useful anticancer medicines.

## Figures and Tables

**Table 1:** Functional classes of anticancer peptides and their development implications.

Functional Class	Primary Therapeutic Job	Decisive Early Measurements	Recurrent Failure Mode	Product-Development Implication	Common/Optimal Route(s)
Direct tumoricidal/oncolytic	Kill tumor cells through membrane or organelle injury	Kinetics of killing; normal-cell and erythrocyte selectivity; serum effects; local immune consequences	In vitro potency does not survive plasma binding or cannot be separated from host-membrane toxicity	Define local versus systemic use before sequence optimization	Intratumoral or other local/regional delivery; systemic only with a strong safety margin
Intracellular/PPI modulator	Block or restore a disease-driving protein interaction	Target affinity in native context; cell entry; endosomal escape; subcellular exposure; target engagement	Strong biochemical binding but inadequate free intracellular concentration	Optimize affinity, permeability, and metabolic stability together	Intravenous/systemic; local or regional delivery when anatomy permits
Homing/penetrating peptide	Direct or increase delivery to tumor tissue	Receptor density; binding/internalization; spatial tumor distribution; effect on partner-drug exposure	Target heterogeneity or increased delivery to normal tissues	Co-develop peptide with biomarker and intended partner therapy	Intravenous, usually co-administered or conjugated with the partner therapy
Peptide–drug conjugate	Carry a cytotoxic, imaging, or radionuclide payload	Intact-conjugate PK; linker stability; payload release; tissue distribution; bystander effect	Premature release, poor penetration, or conjugate aggregation	Treat peptide, linker, and payload as one new molecular entity	Intravenous/systemic; local delivery may suit selected payloads or indications

**Table 2:** Clinical evidence, current outcomes, and translational lessons from representative peptide programs.

Peptide/Program	Product Role and Route	Original Therapeutic Hypothesis	Clinical Evidence	Current Outcome/Status	Principal Translational Lesson
LTX-315	Direct oncolytic 9-mer; intratumoral	Local membrane disruption can debulk lesions, release antigens and danger signals, and support immune priming.	Phase I showed local injury, increased CD8-positive infiltration, lesion-level activity, and injection-related or systemic reactions [8,9].	Clinical evaluation has remained centered on local administration and combinations.	Route can align exposure with mechanism; lesion-specific and immune endpoints should be prespecified.
p28	Cell-penetrating p53-pathway modulator; intravenous	Preferential tumor-cell entry and p53 stabilization can produce non-lytic antitumor activity.	An initial phase I trial in patients with advanced solid tumors reported tolerability and signals of biological activity [12].	Phase I completed; confirmatory efficacy and biomarker-led development have not been established.	Early systemic safety is feasible, but pathway dependence, productive delivery, and pharmacodynamic selection are essential.
ALRN-6924	Stapled MDM2/MDMX inhibitor; intravenous	A stabilized p53-mimetic helix can enter cells, inhibit MDM2/MDMX, and reactivate wild-type p53.	Phase I demonstrated systemic exposure, target pharmacology, and antitumor activity in a subset [13].	Original systemic development was curtailed; local intraocular evaluation for retinoblastoma was subsequently proposed [14].	Structural stabilization can enable intracellular pharmacology, but genotype, pathway competence, exposure, schedule, and indication must align.
CEND-1 (certepetide)	Cyclic tumor-penetrating peptide; intravenous with chemotherapy	Integrin binding and proteolytic CendR activation can increase partner-drug penetration through neuropilin-1-dependent transport.	Phase I supported combination feasibility [17]; randomized phase II ASCEND meeting reports showed cohort-dependent PFS and response signals [18].	Randomized phase II evaluation completed; full peer-reviewed confirmation and definitive validation remain pending.	An enabling peptide should be judged by incremental partner-drug delivery and randomized benefit over the same backbone.
Cilengitide	Cyclic RGD integrin inhibitor; intravenous	High-affinity inhibition of αvβ3/αvβ5 integrins was expected to disrupt tumor angiogenesis and integrin signaling.	Phase III addition to standard therapy in selected glioblastoma did not improve outcome [38].	Development in glioblastoma was discontinued after the negative randomized trial.	Target affinity and early activity cannot substitute for validated disease biology and exposure-response relationships.
177Lu-DOTATATE	SSTR-targeted radioligand; intravenous; adjacent modality	An SSTR2-binding somatostatin analogue can deliver β-radiation selectively to receptor-positive neuroendocrine tumors.	Randomized phase III NETTER-1 demonstrated efficacy in receptor-positive disease [20].	Established peptide receptor radionuclide therapy for SSTR-positive neuroendocrine tumors.	Peptide targeting is strongest when paired with receptor imaging, patient selection, biodistribution assessment, and payload-specific dosimetry.

**Table 3:** Representative sequences or structural formats and structure–property implications of clinically informative anticancer peptide programs. Exact amino-acid sequences are reported when available from primary public sources; for proprietary or chemically complex candidates, the disclosed structural class and defining chemical features are provided instead.

Program	Public Sequence or Structural Notation	Defining Chemical Features	Structure–Property and Design Implication
LTX-315	K–K–W–W–K–K–W–Dip–K–NH_2_ [8]	Nine-residue C-terminal amide containing the non-canonical aromatic residue β-diphenylalanine (Dip); strongly cationic and amphipathic.	The Lys/aromatic balance supports rapid membrane disruption and local oncolysis, but the same profile can increase hemolysis, aggregation, serum binding, and nonspecific tissue interactions.
p28	LSTAADMQGVVTDGMASGLDKDYLKPDD [12]	Linear 28-residue azurin fragment (residues 50–77); water-soluble, amphipathic, and net anionic; no macrocyclic constraint.	The native sequence supports preferential cellular entry and p53-pathway modulation; linearity preserves conformational flexibility but creates proteolytic and exposure liabilities.
ALRN-6924	17-residue p53-mimetic stabilized α-helix; residue-level representation requires non-canonical staple notation [13,39]	All-hydrocarbon-stapled peptide with non-canonical olefin-bearing residues; a long-acting active intracellular metabolite has been described.	Helical preorganization improves dual MDM2/MDMX affinity, protease resistance, and cellular access, but staple placement and metabolism can alter solubility, exposure, affinity, and safety.
CEND-1	c(CRGDKGPDC), with a Cys1–Cys9 disulfide [16,17]	Cyclic nine-residue iRGD peptide containing an RGD integrin-binding motif and a cryptic CendR motif exposed by proteolysis.	Cyclization supports stability and integrin recognition; productive penetration depends on cleavage, neuropilin-1 engagement, receptor distribution, and route-matched exposure.
Cilengitide	c(RGDf(NMe)V): cyclo[Arg–Gly–Asp–D-Phe–N-methyl-Val] [38,40]	Head-to-tail cyclic pentapeptide incorporating D-Phe and N-methylation.	Cyclization and N-methylation rigidify the RGD pharmacophore and improve proteolytic stability and αv-integrin affinity, but affinity alone did not ensure clinical efficacy.
177Lu-DOTATATE	177Lu-DOTA–D-Phe–c[Cys–Tyr–D-Trp–Lys–Thr–Cys]–Thr; Cys2–Cys7 disulfide [20,41]	Cyclic octreotate containing D-amino acids, an N-terminal DOTA chelator, and the β-emitting 177Lu payload.	The constrained somatostatin analogue provides SSTR2 recognition, whereas radiochemistry, receptor density and internalization, renal distribution, and dosimetry determine the complete product profile.

Abbreviations and notation: c, cyclic peptide; Dip, β-diphenylalanine; DOTA, 1,4,7,10-tetraazacyclododecane-1,4,7,10-tetraacetic acid; f, D-phenylalanine; NMe, N-methyl; SSTR2, somatostatin receptor subtype 2.

## Data Availability

No new data were created or analyzed in this study. Data sharing is not applicable to this article.
